# Extracellular vesicles in diagnostics and therapy of the ischaemic heart: Position Paper from the Working Group on Cellular Biology of the Heart of the European Society of Cardiology

**DOI:** 10.1093/cvr/cvx211

**Published:** 2017-11-02

**Authors:** Joost Petrus Gerardus Sluijter, Sean Michael Davidson, Chantal M Boulanger, Edit Iren Buzás, Dominique Paschalis Victor de Kleijn, Felix Benedikt Engel, Zoltán Giricz, Derek J Hausenloy, Raj Kishore, Sandrine Lecour, Jonathan Leor, Rosalinda Madonna, Cinzia Perrino, Fabrice Prunier, Susmita Sahoo, Ray Michel Schiffelers, Rainer Schulz, Linda Wilhelmina Van Laake, Kirsti Ytrehus, Péter Ferdinandy

**Affiliations:** 1 Experimental Cardiology Laboratory, UMC Utrecht Regenerative Medicine Center, University Medical Center Utrecht, University Utrecht, Utrecht, The Netherlands; 2 The Hatter Cardiovascular Institute, University College London, London, UK; 3 INSERM UMR-S 970, Paris Cardiovascular Research Center–PARCC, Paris, France; 4 Department of Genetics, Cell- and Immunobiology, Semmelweis University, Budapest, Hungary; 5 MTA-SE Immunoproteogenomics Research Group, Budapest, Hungary; 6 Department of Vascular Surgery, UMC Utrecht, Utrecht University, Utrecht, the Netherlands; 7 Netherlands Heart Institute, Utrecht, the Netherlands; 8 Experimental Renal and Cardiovascular Research, Department of Nephropathology, Institute of Pathology, Friedrich-Alexander-Universität Erlangen-Nürnberg, Erlangen, Germany; 9 Department of Pharmacology and Pharmacotherapy, Semmelweis University, Budapest, Hungary; 10 Cardiovascular & Metabolic Disorders Program, Duke-National University of Singapore Medical School, 8 College Road, Singapore; 11 National Heart Research Institute Singapore, National Heart Centre Singapore, 5 Hospital Drive, Singapore; 12 Yong Loo Lin School of Medicine, National University Singapore, 1E Kent Ridge Road, Singapore; 13 The Hatter Cardiovascular Institute, University College London, 67 Chenies Mews, London, UK; 14 The National Institute of Health Research University College London Hospitals Biomedical Research Centre, Research & Development, Maple House 1st floor, 149 Tottenham Court Road, London, UK; 15 Department of Cardiology, Barts Heart Centre, St Bartholomew's Hospital, W Smithfield, London, UK; 16 Department of Pharmacology, Center for Translational Medicine, Lewis Katz School of Medicine, Temple University, Philadelphia, PA, USA; 17 Hatter Institute for Cardiovascular Research in Africa and Lionel Opie Preclinical Imaging Core Facility, Faculty of Health Sciences, University of Cape Town, South Africa; 18 Neufeld Cardiac Research Institute, Sackler Faculty of Medicine, Tel-Aviv University, Tel Hashomer, Israel; Tamman Cardiovascular Research Institute, Heart Center, Sheba Medical Center, Tel Hashomer, Israel; 19 Center of Aging Science and Regenerative Medicine, CESI-Met and Institute of Cardiology, “G. D'Annunzio” University, Chieti-Pescara, Chieti, Italy; 20 Department of Internal Medicine, University of Texas Medical School in Houston, TX, USA; 21 Texas Heart Institute, Houston, TX, USA; 22 Department of Advanced Biomedical Sciences, Federico II University, Naples, Italy; 23 Institut Mitovasc, CHU d’Angers, Université d’Angers, Angers, France; 24 Cardiovascular Research Institute, Icahn School of Medicine at Mount Sinai, New York, NY, USA; 25 Laboratory Clinical Chemistry and Hematology Division, University Medical Center Utrecht, Utrecht, The Netherlands; 26 Institute of Physiology, Justus-Liebig University of Giessen, Aulweg 129, Giessen, Germany; 27 Division Heart and Lungs, and Hubrecht Institute, University Medical Center Utrecht, Utrecht, The Netherlands; 28 Cardiovascular Research Group, Department of Medical Biology, UiT The Arctic University of Norway, Tromsø, Norway; 29 Department of Pharmacology and Pharmacotherapy, Semmelweis University, Nagyvárad tér 4, Budapest, Hungary and; 30 Pharmahungary Group, Szeged, Hungary

**Keywords:** Exosomes, Microvesicles, Extracellular vesicles, Ischaemia, Reperfusion, Cardioprotection, Heart failure, Remote conditioning, Preconditioning, Postconditioning, Co-morbidities, Regenerative medicine

## Abstract

Extracellular vesicles (EVs)—particularly exosomes and microvesicles (MVs)—are attracting considerable interest in the cardiovascular field as the wide range of their functions is recognized. These capabilities include transporting regulatory molecules including different RNA species, lipids, and proteins through the extracellular space including blood and delivering these cargos to recipient cells to modify cellular activity. EVs powerfully stimulate angiogenesis, and can protect the heart against myocardial infarction. They also appear to mediate some of the paracrine effects of cells, and have therefore been proposed as a potential alternative to cell-based regenerative therapies. Moreover, EVs of different sources may be useful biomarkers of cardiovascular disease identities. However, the methods used for the detection and isolation of EVs have several limitations and vary widely between studies, leading to uncertainties regarding the exact population of EVs studied and how to interpret the data. The number of publications in the exosome and MV field has been increasing exponentially in recent years and, therefore, in this ESC Working Group Position Paper, the overall objective is to provide a set of recommendations for the analysis and translational application of EVs focussing on the diagnosis and therapy of the ischaemic heart. This should help to ensure that the data from emerging studies are robust and repeatable, and optimize the pathway towards the diagnostic and therapeutic use of EVs in clinical studies for patient benefit.

## 1. Introduction

### 1.1 Cellular secretion for communication; extracellular vesicles

**Figure 1:**
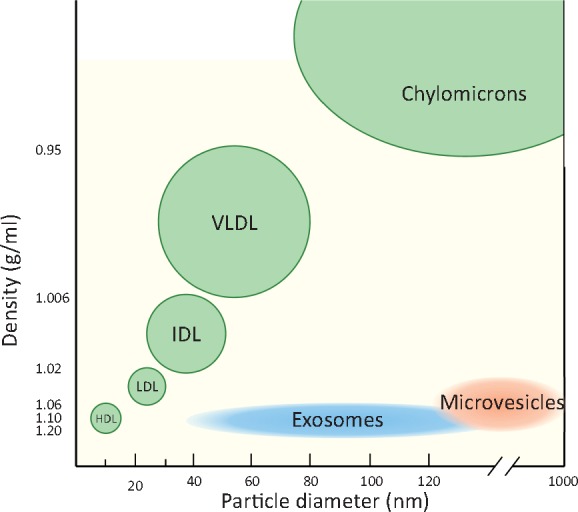
Exosomes and MVs overlap in size with VLDL and chylomicrons, and in density with HDL/LDL particles. Exosome density is typically 1.06–1.20 g/mL. MV density is not well defined but they have been found between ∼1.03–1.08 g/mL.^10–12^

Cells in multicellular organisms must communicate efficiently with each other in order to propagate signals and co-ordinate function. In addition to distinct chemical signals (paracrine and endocrine) and direct cell-cell contact, a growing body of evidence shows that cells communicate via a variety of small, membrane-enclosed vesicles, collectively termed ‘extracellular vesicles’ (EVs). EVs ranging from ∼40 nm to several microns in size (*Figure 1*), are released into all extracellular fluids including blood. They transport a cell-specific cargo of proteins, lipids, metabolites, and nucleic acids that can affect target cells. This process occurs during normal cellular physiology as well as during stress and disease. In this rapidly evolving area of research, with its associated technical challenges, it is vital to establish standardized techniques for their isolation and criteria for their identification. EVs are released by all cardiac, endothelial and inflammatory cell types, suggesting they have an important role in the cardiovascular system, including the ischaemic heart.^1–6^

Therefore, the overall objective of this position paper is to provide a set of recommendations for the isolation, characterization, analysis, and translational application of EVs focussing mainly on the ischaemic heart, i.e. acute myocardial infarction and post-ischaemic heart failure. Coronary atherosclerosis is not discussed in this position paper in detail (see recent reviews and position papers of the European Society of Cardiology that covers this topic extensively^7–9^).

## 2. Isolation and characterization of EVs

### 2.1 Definition of EVs

**Figure 2:**
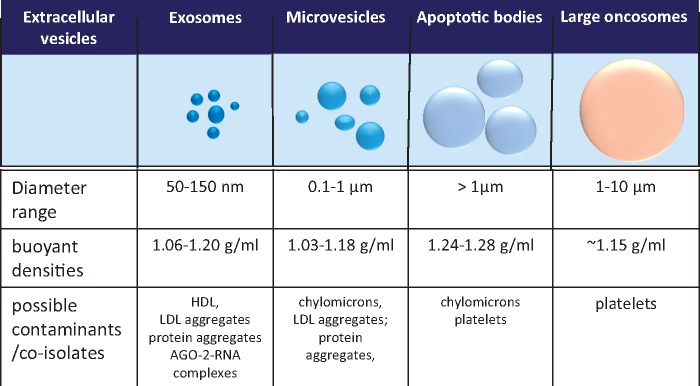
The major classes of EV. Typical size and density of EV classes and some of the contaminants that may be co-isolated, depending on biofluid.

Eukaryotic EVs include (i) exosomes released by exocytosis of multivesicular bodies (usually 50–150 nm), (ii) Microvesicles (MVs, also called microparticles or ectosomes), vesicles around 0.1–1 μm in diameter shed from the plasma membrane,^10–12^ and (iii) apoptotic vesicles released by blebbing of apoptotic cells some of which are > 1 μm (apoptotic bodies),^13^^,^^14^ and (iv) large oncosomes released by migratory tumour cells (> 1 µm)^15^^,^^16^ (*Figure 2*). However, since current isolation protocols only result in relative enrichment of vesicle subpopulations rather than their complete purification,^17^ and that no specific markers are available for the subpopulations, it is preferable to refer to purified vesicles as ‘EVs’ and accurately report the purification method used and characteristics present. An operational definition of small EVs (sEVs) is appropriate for the exosome-enriched population pelleting at high speeds.^17^

### 2.2 Methods for the isolation and characterization of EVs

**Figure 3:**
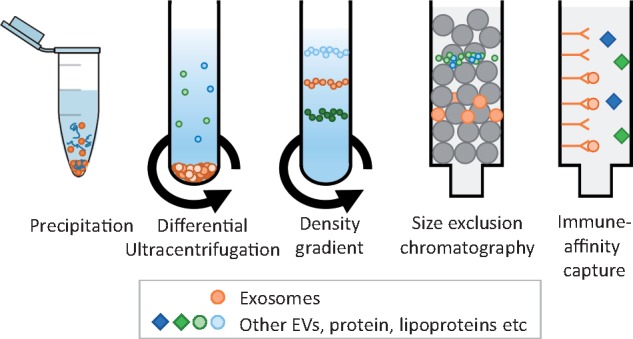
Standard techniques used for isolating exosomes from other EVs, protein, and lipoproteins present in blood and cell-culture medium.

Several methods of isolating EVs have been developed (*Figure 3*). The optimal EV isolation procedure will depend on the source biofluid, the EV subpopulation of interest, and their intended end-use, whether that is to be diagnostic biomarker studies, mechanistic studies, or for *in vivo* administration. However, no EV isolation method yet exists that can be considered as a gold standard, since residual proteins and/or lipoproteins remains problematic.^18^ Complete removal of lipoproteins (present in both blood and tissue culture serum) remains challenging due to overlapping size and/or densities between EVs and different lipoprotein particles (*Figures 1* and *2*).^12^^,^^19^^,^^20^ Moreover, low density lipoprotein (LDL) and exosomes may associate, rendering their complete separation from blood samples impossible using any technique.^12^ This, however, might be used in isolating a subset of EVs co-precipitated with LDL or high density lipoprotein (HDL) particles.^21^^,^^22^

In the simplest technique based on precipitation (e.g. ExoQuick^TM^), the biofluid sample is mixed and incubated with a hydrophilic polymer prior to low speed centrifugation. The polymers attract water molecules away from the solvation layer around the EVs causing their precipitation. Several manufacturers now market products based on this technique. However, they result in high levels of contaminants including serum proteins and lipoproteins as well as residual matrix that can affect EV biological functions.^23^^,^^24^

Differential centrifugation has long been regarded as the gold standard technique.^19^ In the most commonly used protocol, cells are removed by centrifugation at 300 x g for 10 min; the supernatant is then cleared of apoptotic bodies by centrifugation at 2000 x g for 10 min followed by 10 000 x g for 30 min to preferentially pellet MVs; exosome-enriched sEVs are then purified from the supernatant by ultracentrifugation at 100 000 x g for 70 min.^19^ However, optimal parameters are highly dependent on the type of centrifuge rotor used.^25^ For further purification of EVs from co-pelleted protein complexes^26^ and lipoproteins, a density gradient is recommended (sucrose or preferably iodixanol).^19^ Because of the number of steps involved and the length of the procedure, it is clearly unsuited for the analysis of large numbers of samples. More recently, concerns have been raised that ultracentrifugation can cause damage, fusion, and/or aggregation of vesicles.^27^

There is increasing enthusiasm for the use of size-exclusion chromatography.^28^ Suitable matrices include Sepharose 2B, Sepharose CL-4B, or Sephacryl S-400.^29^ The resolution of size separation is dependent on column length. This technique is effective at separating EVs from proteins and some lipoproteins, but samples usually become considerably diluted and efficient separation of lipoproteins remain challenging.^12^^,^^29^^,^^30^

Other techniques are less well established. Filtration (0.2 μm–0.8 μm) aids in removal of larger vesicles, but under high pressure it is possible that it could cause the fragmentation of larger EVs into smaller vesicles. Immuno-affinity can be an effective means to isolate specific EV populations, using columns or magnetic beads for example, but for functional follow-up studies this is still challenging. Flow cytometry-based analysis of individual EVs is a valuable tool for EV characterization and quantification, although it remains challenging due to size and sensitivity limitations.^31^ Progress has been made in developing these technologies for specific EV sorting, but some pre-requisite steps need to be taken.^32^ Furthermore, this approach requires certainty as to the specificity of epitopes expressed by the desired population of EVs–which may not be the case for exosomes.^33^ Other isolation techniques such as microfluidics are under development but have not yet been rigorously tested. For pre-clinical or clinical studies of the ischaemic heart, measurement of EVs can be performed from *in vivo* blood, lymphatic or pericardial fluid samples, *ex vivo* heart perfusate samples, and tissue culture media samples that may require different isolation techniques.

#### 2.2.1 Isolation from blood

Pre-analytical procedures can have a large impact on blood EV measurements. For example, since clotting may increase the number of EVs in blood by 10-fold,^34^ it is usually preferable to use plasma. On the other hand, serum may be useful when overall yield of platelet MVs is more important than accurate quantification of particle number. A crucial concern is the minimization of platelet activation and EV release. Standardized procedures to minimize platelet activation during plasma isolation should be followed.^35^^,^^36^ Fasting before blood sampling can help to minimize chylomicron contamination.^12^ Blood should be collected in citrated or acid-citrate-dextrose anticoagulant tubes,^23^^,^^35^^,^^37^ such as vacutainers, and the first tube of blood should be discarded.^23^^,^^35^ It is recommended to dilute blood plasma or serum at least 2x in Ca^2+^-free phosphate buffered saline (PBS) prior to centrifugation in order to reduce the viscosity.^19^ However, if annexin V binding will be assessed (which requires Ca^2+^), PBS should be avoided in order to prevent formation of calcium-phosphate micro-precipitates. The plasma or serum should be centrifuged within 2 h, and agitation avoided.^35^^,^^38^ After centrifugation at 2500 x g for 15 min at room temperature without application of the centrifuge brake, plasma can be carefully collected, and re-centrifuged under identical conditions. This platelet-free-plasma may be snap frozen and stored at –80 °C prior to analysis.

Even when using the same protocol, inter-laboratory variability in plasma EV counts can vary by an order of magnitude.^35^ Given these problems of irreproducibility, The International Society on Thrombosis, and Haemostasis has advised that further refinements are required before flow cytometric enumeration of platelet MV numbers is ready for clinical use.^35^

#### 2.2.2 Isolation from pericardial fluid

Pericardial fluid contains EVs that may provide useful biomarker information about cardiac health.^39^^,^^40^ As yet there is no consensus as to the ideal method for isolation of EVs from pericardial fluid.

#### 2.2.3 Isolation from conditioned media of cultured cells

For the isolation of vesicles produced by cells in tissue culture the important considerations are quite different. The main potential source of contamination is typically from foetal calf serum (FCS) added to the culture medium.^41^ FCS contains large number of vesicles including exosomes as well as lipoproteins. Exosomes can be largely removed by pre-treating FCS by 18 h ultracentrifugation at 100 000 × g,^41^ and removal is enhanced by diluting FCS five-fold in culture medium to reduce viscosity.^23^ Several companies market FCS which has been processed to remove exosomes, though the method used is not specified. However, some caution should be taken for FBS-associated RNA which might be co-isolated with cell-culture derived extracellular RNA (exRNA), thereby interfering with the downstream RNA analysis.^42^ Alternatively, pre-defined serum or serum-free conditions can be used, and indeed is essential if preparing EVs for clinical use.^43^ However, cells may undergo apoptosis or autophagy and release apoptotic bodies after extended periods in the absence of serum. Conditioned medium is usually collected after 24–48 h culture. Although sequential filtration offers the advantage of using large volumes of culture media,^44^ its effect on biological activity of the isolated EVs has not been well characterized. HPLC has been successfully used to purify exosomes.^45^

#### 2.2.4 Isolation from isolated heart perfusate

EVs can be isolated from hearts perfused with buffer such as those mounted on a Langendorff apparatus.^46^ Pre-concentration of the perfusate by ultrafiltration may be necessary for a sufficient yield, but subsequently any of the techniques described above may be used. It is important to be aware that exosome-sized, calcium-phosphate nanoparticles form spontaneously in Ca^2+^-containing bicarbonate buffer, which can interfere with some analyses such as nanoparticle tracking analysis.^47^

#### 2.2.5 Storage of EVs

EVs appear to be relatively stable when stored at –80 °C or less,^48^ but repeated freeze-thaw cycles should be avoided and cryo-preservatives such as glycerol and DMSO should not be added as they may lyse EVs.^48^ Trehalose has recently been proposed to preserve exosomes.^49^

#### 2.2.6 Visualization and quantitation of EVs

**Figure 4:**
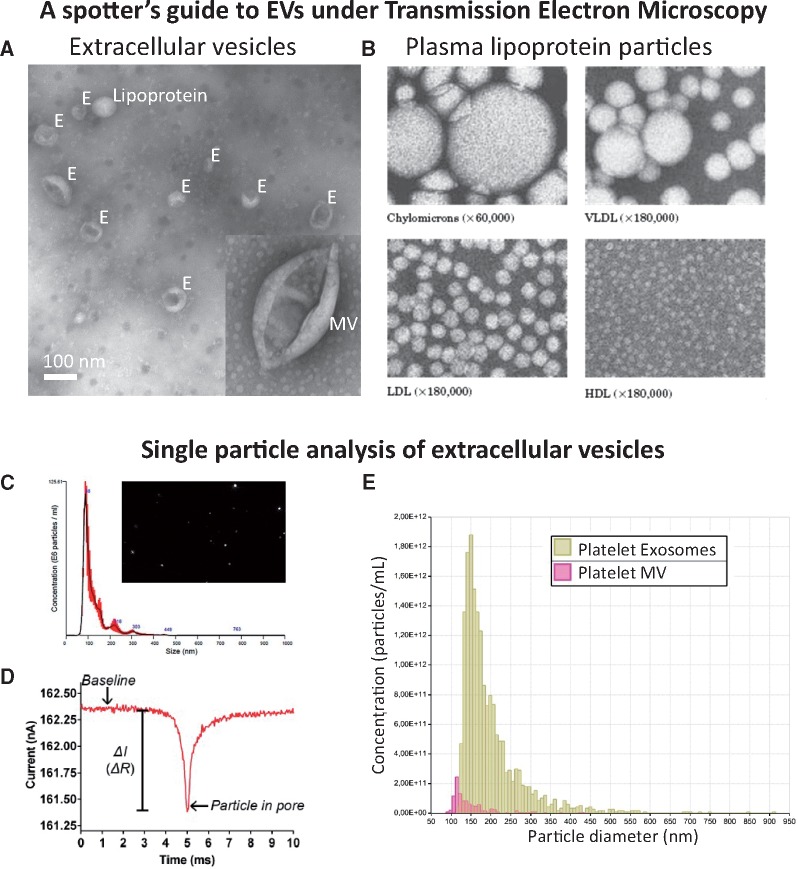
Standard methods of characterizing EVs. (*A*) Transmission electron microscopy (TEM) of negative-stained EVs reveals the ‘cup-shaped’ appearance of exosomes (E) and MVs once they have been dried for TEM (they are spherical in solution). (*B*) The spherical appearance of lipoprotein particles by TEM is quite distinct (image courtesy of Robert L. Hamilton and the Arteriosclerosis Specialized Center of Research, University of California, San Francisco). (*C*) Nanoparticle tracking analysis (NTA) provides a size distribution of particles based on calculating their size by their random Brownian motion. (*D*, *E*) Tuneable resistance pulse sensing (TRP) determines size distribution by the change in resistance as the particle crosses a small pore in a membrane (which is selected according to the size range examined).

In most studies, it is informative to quantify EVs using either nanoparticle tracking analysis, tuneable resistive pulse sensing, or dynamic light scattering (*Figure 4*). Importantly, these methods usually do not discriminate EVs from non-vesicular events such as LDL particles.^12^ The ratio of particle number or lipid content to protein content of the preparations can give an indication of relative EV purity, as compared to protein concentration only.^50^^,^^51^ Exosome morphology should be confirmed by transmission electron microscopy,^19^ cryo-electron microscopy,^52^ or atomic force microscopy.^53^ In addition, presented images should contain multiple EVs per field (*Figure 4A*). Using flow cytometry, the lower size-limit of detection is steadily decreasing, but it remains technically challenging to obtain comprehensive analyses of MVs let alone exosome-sized particles.^32^

#### 2.2.7 Characterization of EVs

Further characterization of EVs should include the detection of specific markers. It is generally recommended to demonstrate presence of tetraspanin proteins such as CD9, CD81, CD63, and the intra-vesicular protein Alix, which are involved in exosome biogenesis, in addition to other typical marker proteins such as HSP70, flotillin-1, or major histocompatibility complex (MHC) class I and class II. However, recent studies indicate some of these are not as specific for exosomes as thought.^17^^,^^54^ Co-enrichment of CD63, CD9, and CD81 tetraspanins and endosome markers such as syntenin-1 and TSG101 may be seen as indicative of exosome presence.^17^ MV surface marker expression is a useful index of cell-type of origin, and can be quantified by flow cytometry in order to analyse sub-populations. In addition, MVs may be characterized as ‘calcium-dependent annexin V binding’ or ‘calcium-independent lactadherin (MFGE8) binding’. Gating strategies for flow cytometry involve the use of fluorescent reference beads of known sizes. Preferred beads are the silica beads because their refractive index is close to the one of biological particles.^55^ Of note, not all MVs are detectable this way. To quantitatively detect exosome proteins, a nano-plasmonic exosome sensor was developed that comprises arrays of periodic nanoholes patterned in a metal film. The arrays are functionalized with affinity ligands for different exosomal protein markers and offers highly sensitive and label-free exosome analyses by continuous and real-time monitoring of molecular binding.^56^

In order to demonstrate EV functionality it may be useful to demonstrate their interaction with, fusion, or uptake into recipient cells. To this end, EVs can be fluorescently labelled with lipophilic dyes, or by transfection of parent cells with GFP-tagged proteins packaged in EVs. Control experiments using ‘dye-only’ samples prepared in parallel the same way as EVs are essential to confirm the involvement of EVs^57^ and that free dye has been removed, which can otherwise form micelles. Density gradient ultracentrifugation is a frequently used approach to remove this non-EV-associated dye. Co-incubation with receptor antagonists or uptake inhibitors will provide insights in mechanisms of uptake although these compounds are notorious for their toxicity and limited specificity.^58^

#### 2.2.8 Analysis of nucleic acid, protein, and lipid contents of EVs

Proteomics can provide a more comprehensive description of EV protein content, but the isolation protocol must be carefully optimized in order to remove plasma protein contamination in blood EV samples.^59^^,^^60^ Similar concerns arise with transcriptomic analyses of miRNA, mRNA, or DNA contents, particularly with regard to potentially contaminating HDL particles and argonaute 2-RNA complexes, which are known to transport miRNA.^61^ Care must be taken to avoid haemolysis which can affect circulating miRNA levels.^62^

EV proteomics has been studies in large clinical cohorts,^63^ but undoubtedly the greatest interest is currently focused at the RNA content of EVs as potential biomarkers.^63^EV lipidomics is an interesting but under-explored area.^64^ Nucleic acid, protein and lipid entries from EV studies are available in EV databases (http://www.exocarta.org/, http://student4.postech.ac.kr/evpedia2_xe/xe/ and http://microvesicles.org/ (10 November 2017, date last accessed)). However, because of the diversity of EV isolation protocols used, these database entries should be treated with caution.

#### 2.2.9 Technical control experiments

RNase or protease may be applied to remove extra-vesicular nucleic acids and proteins and confirm intra-vesicular localization. Detergent control during flow cytometry or sizing performed by tuneable resistive pulse sensing or dynamic light scattering enables distinguishing EVs from protein aggregates (the latter being more resistant to detergents lysis than EVs).^26^^,^^65^^,^^66^ Of note, small and large EVs have different detergent sensitivities^67^ and lipoproteins also show a limited sensitivity to detergent lysis.^12^

### 2.3 Recommendations for isolation and purification of EVs

At present, there is no universally agreed protocol for isolation of pure populations of EVs or subpopulations of EVs. Even their precise nomenclature is in flux, and will presumably remain so until clear surface protein signatures of individual EV subtypes will be established. Given the rapid developments in this area it is important to remain cognizant of experimental limitations and caveats in order to avoid overstating conclusions. At present, it is challenging to isolate EVs from tissue homogenates. The likely contamination of EV preparations with proteins and lipoproteins renders many results suspect unless crucial control experiments are performed. Differential detergent lysis is a simple, inexpensive EV control for flow cytometry.^26^^,^^65^ Furthermore, to avoid swarm detection at high particle concentrations, analysis of serial dilution of samples is recommended during flow cytometry.^32^ A characterization of EV proteins will provide a basis on purity without excluding a potential biological role. *Table 1* provides a check-list of criteria for the successful isolation and characterization of EVs, as well as more specific criteria for exosomes.

**Table 1:** Recommendations for the isolation and characterization of EVs [adapted from Ref. 18]

**General recommendations for EVs**
EVs can be isolated from either tissue culture supernatant or extracellular fluids. Reliable methods for EV isolation from tissue homogenates remain to be established. Ensure consistency of pre-analytical procedures. Report complete experimental details, including pre-analytical and isolation procedure and details of all antibodies used.^190^ Also include all negative data sets. If EV function is analysed, include: a dose-response curve. systematic negative (‘EV-depleted’) controls. demonstrate an association between a protein/miRNA and EVs in support of any function ascribed to them, e.g. using immuno-EM, or co-purification on a density gradient.
**Specific recommendations for exosomes**
Avoid precipitation methods of isolation, Tissue culture of cells for exosome isolation must be cultured with exosome-free FCS or under FCS-free conditions, Confirm the presence of at least 3 ‘marker’ proteins typically enriched in exosomes, e.g. Tetraspanins: CD9, CD63^a^, and CD81 Endosomal markers: Syntenin-1 and TSG101 Assess levels of contaminating proteins, e.g. serum albumin, extracellular matrix, mitochondrial, nuclear protein, argonaute, lipoproteins. It is not currently possible to state a ‘minimum acceptable level’ but protein contamination can form an important internal quality control. If electron microscope images are shown, they should include more than 1 exosome per field. Determine the size distribution using two orthogonal techniques, e.g.: nanoparticle tracking analysis, electron microscopy, tuneable resistive pulse sensing or dynamic light scattering.
**Specific recommendations for MVs**
Establish rigorous guidelines for consistency of isolation methods, Determine the accuracy and precision (coefficient of variation) of the quantification methods used.

a Some markers such as CD63 may not be completely specific for exosomes.^17^

## 3. Mechanism of EV actions in the heart

EVs appear to be released from all major cell types found in the heart. For example, exosomes have been shown to be released by primary adult cardiomyocytes,^68^^,^^69^ primary cardiac endothelial cells,^70^ primary cardiac fibroblasts,^71^ and vascular smooth muscle cells.^72^ Most MVs present in the plasma of healthy individuals are derived from platelets and erythrocytes, but plasma MVs also originate from leucocytes, endothelial cells, monocytes neutrophils, and lymphocytes.^73^^,^^74^ Most mechanistic experiments to date use EVs isolated from cultured cells, since isolation of cell-type specific EVs *in vivo* is not currently feasible. Recently, a clear overview was provided on the role of EVs in coronary artery disease.^7^ This section will discuss the most relevant examples of the function of EVs released from cells of the heart tissue and their postulated mechanisms of action.

### 3.1 Cardiomyocyte-derived EVs

Cardiomyocytes are potentially an important source of diagnostic EVs particularly in situations of stress such as myocardial ischaemia and failure.^1^ However, few studies have examined exosomes from adult as opposed to neonatal cardiomyocytes, and even here it is unclear to what extent this relates to the situation *in vivo*. *In vitro*, hypoxia and re-oxygenation leads to the release of heat shock proteins (HSPs) HSP70 and HSP90, as well as HSP60, a ‘danger signal’, via exosomes in primary adult rat cardiomyocytes.^68^ Cardiomyocytes also release EVs containing tumour necrosis factor (TNF)-α,^75^ potentially participating in the propagation of an inflammatory response. Glucose deprivation induced the loading of neonatal rat cardiomyocyte exosomes with functional glucose transporters and glycolytic enzymes. When internalized by endothelial cells, these exosomes increased glucose uptake, glycolytic activity, and pyruvate production in recipient cells.^76^ The transfer of exosomes from glucose-deprived H9C2 cardiac myoblasts to endothelial cells also induced changes in transcriptional activity of pro-angiogenic genes.^77^ Hyperglycaemia altered cardiomyocyte-derived exosomes in a model of diabetes-associated cardiomyopathy,^78^ as well as from the hypoxic myocardium which can activate *in vitro* cultured endothelial cells.^79^^,^^80^ Finally, external stretch caused cardiomyocytes to release exosomes enriched in functional angiotensin II type-1 receptors (AT1Rs).^69^ Administration of AT1R-enriched exosomes restored responsiveness to angiotensin II in the vessels of AT1R-KO mice. Together, these data indicates that cardiomyocytes respond to environmental changes by releasing specific EVs that specifically modulate neighbour-cell function.

### 3.2 Cardiac progenitor cells-derived EVs

A variety of cells with the capacity to proliferate and differentiate into cardiac cells have been termed cardiac progenitor cells (CPCs). Exosomes released from these cell types appear to recapitulate the cardioprotective and regenerative benefits of their parent cells.^81^^,^^82^ Hypoxia stimulated exosome release from CPCs and upregulated their expression of pro-angiogenic genes, anti-fibrotic genes, and a cluster of miRs, and increased their ability to improved cardiac function after IR injury in rats.^83^^,^^84^ Vrijsen *et al.* reported that human CPCs release exosomes into their environment, and that exosomes from CPCs are able to stimulate the migration of endothelial cells in an *in vitro* scratch wound assay.^85^ They also showed that CPC-exosomes contain matrix metalloproteinases (MMPs) and the extracellular matrix metalloproteinase inducer (EMPRINN), which mediate their angiogenic potential.^86^

### 3.3 Endothelial cell-derived EVs

Endothelial cells are an important source of EVs. Vascular endothelial cells secrete exosomes and MVs that exchange biological messages with other cell types of the heart.^4^^,^^7^ There is extensive literature demonstrating the effects of endothelial MV in promoting angiogenesis (reviewed in Ref. 87). Endothelial exosomes have also been shown to stimulate angiogenesis via a mechanism that is believed to involve transfer of miR-146a.^88^ Hypoxia alters mRNA and protein composition of exosomes released by cultured endothelial cells.^89^ TNF-α-treated endothelial cells release exosomes expressing increased levels of intercellular adhesion protein (ICAM)-1.^89^ These findings exemplify the potential function of endothelial cell-derived exosomes and MV that may also make them useful as biomarkers of cardiac stress and disease.^4^^,^^9^

### 3.4 Vascular progenitor cell-derived EVs

While their exact differentiation potential has been debated, it is evident that CD34^+^ cells from bone marrow secrete exosomes that possess angiogenic characteristics, enhance tube formation of endothelial cells, and increase neovascularization *in vivo*.^90^ Further analysis revealed enrichment of several pro-angiogenic miRs (miR-126 and 130a) in CD34^+^ cell-derived exosomes. MVs derived from human vascular progenitor cells carry several markers similar to receptors expressed on their membranes.^91^ In various disease models, these MVs were shown to enhance expression of angiogenic miRs (miR-126 and 296), and promote neovascularization of pancreatic islets^92^ and ischaemic hind limb.^93^

### 3.5 EVs from fibroblasts, smooth muscle cells, and mesenchymal stromal cells

Ischaemia, pressure, and volume overload induce hypertrophic cellular responses mediated by cross-talk among fibroblast cardiomyocytes, endothelial cells, and inflammatory cells via EVs. In response to angiotensin II, cardiac fibroblasts secreted exosomes that stimulated angiotensin II production and its receptor expression in cardiomyocytes, and stimulated myocyte hypertrophy.^94^ Exosomes released from cardiac fibroblasts contained high levels of miR-21-3p/miR-21, which induced cardiomyocyte hypertrophy.^94^ Smooth muscle cells also release exosomes and are implicated in vessel calcification and atherosclerosis.^72^^,^^95^ Mesenchymal stromal/stem cells (MSCs) are resident in almost all tissues, including the heart, and play a major role in tissue repair and regeneration.^96^ Characterization of the MSC exosome proteome has revealed many cytokines, growth factors, inflammatory molecules, components of the extracellular matrix, and proteases. Analysis of protein content revealed >400 different proteins.^97^ Many signalling molecules related to MSC self-renewal, differentiation, and signalling pathways were found to be enriched in MSC exosomes, potentially affecting a diverse range of cellular processes, including cell cycle, proliferation, cell adhesion, cell migration, and cell morphogenesis. Similarly, miRNAs shuttle within MSC exosomes but mostly in precursor form, driving downstream signalling pathways. Additionally, MSC exosomes possess immunologic properties, including secretion of anti-inflammatory cytokines, such as interleukin-10, tumour growth factor (TGF)-β, and promote inhibition of lymphocyte proliferation.^98^

### 3.6 Immune cell-derived EVs

Immune system cells, such as B cells and dendritic cells, mediate MHC-dependent immune responses upon EV secretion.^99^^,^^100^ For this purpose, vesicles express particular adhesion molecules for specific targeting of recipient cells.^101^ Other immune cells release MVs with immune functions, for example, NK-derived exosomes enclose perforin and granzyme B and mediate anti-tumour activities either *in vitro* or *in vivo*.^102^ Furthermore, peptides expressed in exosomes released by mast cells are presented by DCs and induce specific immune responses *in vivo*.^103^ It has also been reported that macrophages release IL-1β on inflammasome activation, suggesting that these MVs play a role in pro-inflammatory activity and innate immune response.^104^

### 3.7 Platelet-derived EVs

Platelets release both exosomes and MVs,^105^ and release is strikingly enhanced by many stimuli, including physicochemical stresses and apoptosis.^106^ Platelet-derived exosomes are able to regulate the coagulation response,^105^ and mediate platelet atherogenic interactions with endothelial cells and monocytes.^107^^,^^108^ Platelet MV stimulate angiogenesis and intramyocardial injection improved post-ischaemic revascularization.^109^ Platelet exosomal cargoes include diverse cytokines, chemokines, growth factors, coagulation factors, lipoproteins, and other lipids, as well as several types of RNA.^105^^,^^106^^,^^110^^,^^111^ Platelet exosomal membrane proteins also reflect their platelet source, including the constitutively expressed glycoprotein GPIb, as well as GPVI, αIIbβ3, CD40 ligand, and P-selectin from activated platelets.^105^^,^^106^

It has been suggested that platelet activation in some vascular diseases will elevate loading of cyto-adhesive, thrombogenic, and inflammatory factors into platelet exosomal cargo to promote their delivery to endothelial cells and macrophages at sites of vascular lesions. Augmented delivery of platelet exosomal atherogenic cargo to lesional endothelial cells and macrophages, may consequently accelerate development of vascular plaques, clots, and atherosclerosis.^107^^,^^108^^,^^112^

### 3.8 Technical control experiments for mechanistic studies

Given that current methods of purification are imperfect, the inclusion of appropriate control experiments is crucial (*Table 1*). First and foremost, any biological effects observed using purified EVs should be absent in ‘sham’ control samples depleted of EVs. Furthermore, when using EVs isolated from tissue culture medium, inhibition of exosome release may be used to confirm EV involvement in a process (e.g. Rab27a and Rab27b silencing).^113^ Current chemical inhibitors inhibiting sphingomyelinase (e.g. GW4869) are not specific to exosome release.^57^

### 3.9 Recommendations for mechanistic studies

EVs and exosomes appear to mediate a regulated process of intercellular communication, which could be capitalized on in order to obtain information more easily and less invasively than from *in vivo* experiments. However, for studies of exosomal communication, advanced cell models are needed that can compare the use of single cell types with multiple cell types in a 2D or 3D structure. Once identified, exosomal signalling molecules could potentially be used as biomarkers that reflect the cellular state during cardiovascular disease.

Mechanistic experiments should include ‘sham’ EV-depleted control groups. A dose-response curve should be performed to relate the mechanism of action to the EV concentration. Because of the hundreds of different bioactive molecular species in any EV preparation it is often difficult to ascribe function to a specific EV component. At minimum, an association between a protein/miRNA and EVs in support of any function ascribed to them, e.g. using immuno-EM, or co-purification on a density gradient should be performed. It is important to consider the likelihood that a single miRNA or EV component mediates all of its effects, as opposed to there being a network effect of multiple miRNAs, and other mediators.^83^

## 4. EVs as biomarkers for the ischaemic heart

Given their easily-accessible presence in bodily extracellular fluids, EVs have been investigated as potential biomarkers for many diseases, and their presence in blood and pericardial or lymphatic fluid lends promise to their use in ischaemic heart disease. Proteins, lipids, coding and non-coding RNAs, and even DNA specific to their cells of origin are incorporated into EVs and released into bio-fluids. They have therefore been described as a form of ‘liquid biopsy’ of the cell of origin, and its pathophysiological status, and are attractive sources of biomarkers for clinical diagnostic applications in ischaemic heart disease.^114^ Their power derives from the enrichment of potential protein markers which otherwise constitute only a very small proportion (less than 0.01%) of the total proteome of body fluids.^115^ Their sequestration within membranes might also protect the cargo from degradation. Similarly, many miRNAs detectable in serum and saliva are concentrated in exosomes,^116^ where they are protected against RNases.^117^ The RNA content of EVs, especially the miRNA, has provoked great interest as diagnostic biomarkers in exosomal RNA research.^118^ Technical issues related to analysis of EV RNA and proteins contents are described in Section 2.2.8.

Several potential applications for novel biomarkers can be identified. Blood-borne biomarkers of persistent myocardial ischaemia or vascular injury without concomitant cell death are lacking. Subclinical or silent myocardial ischaemia without infarction, different variants of angina and especially microvascular angina^86^ would benefit from additional non-invasive diagnostic options in addition to ECG.^119^ Furthermore, in population studies, biomarkers of persistent low grade myocardial ischaemia without cell death would help to determine the prevalence of such conditions. Similarly, diagnosis of acute coronary syndrome (ACS) need to be improved, especially at early time points after coronary occlusion,^120^ for microvascular angina and in the case of non-ST segment elevation ACS.^121^ Given the wide range of cardiac cell types that are capable of releasing EVs upon exposure towards stress, it is clear that circulating EVs offer great potential for the identification of biomarkers to aid diagnosis. Indeed, circulating MV signature (characterized by e.g. CD66b^+^/CD62E^+^/CD142^+^, or CD235a^+^) in coronary and/or peripheral has been shown to reflect the formation of coronary thrombotic occlusions in STEMI-patients.^122^^,^^123^ Moreover, EVs of lymphatic origin have been recently shown in mice as promising biomarkers for lymphatic dysfunction or inflammatory disease progression in atherosclerosis.^124^

### 4.1 EV number as biomarker

The number of EVs including exosomes seem to be associated with the presence of cardiac ischaemia and correlates with the severity of cardiac injury. The number of procoagulant EVs, particularly MVs, is elevated in the peripheral blood of patients with ACS and chronic ischaemic heart disease.^3^^,^^5^ The quantity of endothelium-derived MVs even allowed discrimination between patients with stable angina, first time-, and recurring myocardial infarction.^125^ Both systemic and intracoronary endothelial and platelet MV correlated with the degree of thrombosis and ischaemic area at risk.^126^^,^^127^ MV protein levels are associated with increased risk for future vascular events and mortality in patients with clinically manifest vascular disease.^128^ In atherosclerosis, increases in EVs of various cell origins have been demonstrated, predicting cardiovascular morbidity and mortality.^129^ However, since reports on both pro- and anti-atherosclerotic effects of different EV populations have been published (see for review: Ref.^130^), their usefulness as diagnostics needs further exploration. Given the caveats mentioned previously with regards the accuracy of current methods of EV quantification, it is important to establish rigorous guidelines for consistency, and to determine the accuracy and precision (coefficient of variation) of the methods used.

### 4.2 EV content as a source of biomarkers

Since EV content is altered by pathology, diagnostics based on the analysis of exosomal content of body fluids may provide further benefit for diagnosing ischaemic heart diseases. The concentration of certain miRNAs in the blood, such as miR-208a, miR-133a, and miR-499 is elevated after ACS.^80^^,^^131^ Circulating miRNA have therefore been proposed as diagnostic biomarkers in cardiovascular diseases.^118^^,^^119^^,^^132–134^ Some of these may be transported by EVs, especially under pathological conditions^80^^,^^116^^,^^135^^,^^136^ or after coronary-artery by-pass graft surgery.^137^ MVs containing miR-126 and miR-199a, but not freely circulating miRNA expression, predict the occurrence of CV events in patients with stable coronary artery disease.^138^ Assessing miRNAs carried specifically by exosomes can even improve diagnostic sensitivity and precision: exosomal miR-208a content correlated well with cTn-I levels after CABG surgery, while the whole-blood miR-208a levels did not.^137^ Similar results were recently reported in ACS patients, where sensitivity of exosomal miR-208a measurement was superior to that of the serum, which even showed a certain degree of prognostic value regarding 1-year survival rate.^139^ Exosomal miR-192, miR-194, and miR-34a have been identified as prognostic biomarkers predicting the development of heart failure and pathological remodelling after myocardial infarction.^140^

The diagnostic value of EV protein content has been little studied. De Hoog *et al.* found that the proteome of ExoQuick^TM^-precipitated EVs from ACS patients differed from those from non-ACS patients: elevated vesicular levels of polygenic immunoglobulin receptor, cystatin C, and complement factor C5a was associated with ACS diagnosis.^141^ Zhang *et al.* showed that protein levels in EV sub-fractions are associated with heart failure in dyspnea cohort.^22^

### 4.3 The effect of co-morbidities on EVs

It is well known that cardiovascular risk factors and co-morbidities (especially aging, gender, and metabolic diseases such as hyperlipidaemia and diabetes), and their medications alter the phenotype of the normal and pathological myocardium including its response to ischaemia/reperfusion and cardioprotective or regenerative therapies (see for extensive reviews).^142–145^ The presence of these confounding factors may interfere with diagnostic opportunities by means of MVs, however, very little is known in this field. Some recent reviews on the diagnosis of diabetes and dyslipidaemia concluded that since EVs may be involved in these pathologies, analysis of EVs might reveal additional biomarkers.^146–148^ For example, the quantity of MVs/microparticles is elevated in the blood plasma of patients with metabolic syndrome,^149^^,^^150^ and elevated levels of certain miRNAs derived from ExoQuick-precipitated EVs have been shown to be associated with metabolic syndrome or diabetes,^151^ but the practical relevance of these reports is yet to be assessed. Since the cardiac transcriptome including miRNA expression profile has been shown to be dramatically changed in the rat myocardium by hyperlipidemia,^152^ analysis of transcriptome including non-coding RNA content of EVs may provide specific diagnostic markers of the heart affected by this and other co-morbidities.^153^ In a study on over 1000 patients, EV-associated cystatin C content was found to be significantly elevated (a marginal, 9% difference), in metabolic syndrome patients with cardiovascular disease, and a proteomics study on EVs derived from adipocytes of obese rats revealed 200 proteins with differential expression.^154^ Moreover, it has been shown that at similar plasma cholesterol levels, patients on statin treatment had a significant lower number of circulating MVs carrying markers of activated cells.^155^ These studies suggest that EVs have the potential to evolve into useful biomarkers of various metabolic diseases in the future, however, a substantial amount of research has to be done before their clinical use might be realized.

### 4.4 Recommendations for study of EVs as biomarkers

Since miRNAs are transferred by either protein complexes, HDL, or EVs,^134^ and since protein contamination of isolated EVs is difficult to control, adequate isolation techniques are of the utmost importance if exosomal miRNAs or proteins are to be assessed as biomarkers. Other recommendations relevant to plasma miRNA biomarker studies in general apply equally to EV miRNA studies.^156^ It should be noted that although detectable amounts of relatively clean EVs can be isolated, e.g. with size exclusion chromatography,^29^ methods for the isolation of EVs need to be improved to allow clinical utilization of EV-based diagnostics.

Current technical limitations for EV isolation do not allow definitive guidelines for the use of EVs as biomarkers. Several factors that are lacking include: the lack of standardized pre-analytical and isolation procedures; lack of gold standard for processing, characterization and purity; an unknown influence of comorbidities, co-medication and other confounding factors; lack of disease specificity; lack of tissue-specific markers (*Table 2*). As with any biomarker study, pilot observations from small cohorts should be validated in larger patient datasets, and the reproducibility of isolation procedures, including markers of EVs and normal range levels in the healthy population should be reported. Moreover, evidence should be provided of the additional value of EVs over current gold-standard biomarkers.

**Table 2:** Current technical limitations for clinical translation of EV biomarker

(1) Lack of standardized pre-analytical and isolation procedures.
(2) Currently no gold standard.
(3) Need to establish methods of purifying specific subpopulations of EVs originating from the heart, vasculature, or blood cells, no golden standard for processing, characterization, and purity.
(4) Unknown influence of confounding factors of EV quality, including disease specificity and presence of comorbidities and their medications.
(5) Small yields of EV subpopulations obtained for content analysis: transcriptomics, lipidomics, and proteomics.
(6) Validation of novel biomarkers in large patient cohorts, including normal range levels in the healthy population.
(7) Determine additional value of EV markers over current golden standard clinical biomarkers, or its use as a combinatory marker.

## 5. Therapeutic potential of EVs for the ischaemic heart

EVs purified from defined cell types have been suggested as novel therapeutic options for various cardiac diseases including ischaemic heart disease, myocardial infarction, and heart failure, as well as for pathogen vaccination, immune-modulatory and regenerative therapies and drug delivery. However, the first clinical steps have been made using exosomes as an anti-tumour therapy: an effective way to destroy non-small cell lung cancer (NSCLC) is to activate tumour-specific cytotoxic T cells (CTL), and for this autologous dendritic cell (DC)-derived exosomes (DEX) loaded with tumour antigens have been tested not only in phase I^157^ but also in a phase II Trial (ClinicalTrials.gov Identifier: NCT01159288). Although this did not induce detectable effector T cell responses, a positive effect on natural killer (NK) cells could be observed in some patients.^158^ This first exosome Phase I trial highlighted the feasibility of large scale exosomes production and the safety of exosome administration.^159^ Several other Phase I studies have been initiated, exploring the use of EVs/exosomes as a therapy, including the effect of MSC-derived exosomes on β-cell mass in Type 1 diabetes mellitus (T1DM) Patients (ClinicalTrials.gov Identifier: NCT02138331).

Little is known about whether EVs derived from animals might be feasible for human therapy. However, EV fractions derived from human umbilical cord MSCs and administered to different healthy and diseased animal species (including rats with myocardial infarction) were found to be well tolerated.^160^ After further study, these results demonstrating a lack of adverse immunological reactions may open the possibility of producing therapeutic EVs from non-human sources. To develop the application of exosome-based therapeutics, a clear understanding of exosome pharmacokinetics *in vivo* is of utmost importance. Currently, only limited information is available, but unmodified EVs derived from tumours are rapidly cleared in liver and spleen by the reticuloendothelial system and cells of the innate immune system.^161^ Interestingly, there may be concerns about intravenous injection of high concentrations of EVs since the authors observed rapid asphyxiation of mice when injecting over 400 µg.^161^ To date, most *in vivo* bio-distribution studies have used small lipophilic fluorescent dyes to label EVs. Although useful for first impressions, the reliability of these analyses might be impaired by the free dye released from EVs.^162^ Bioluminescence imaging of a fusion protein consisting of Gaussia luciferase and lactadherin has been used to demonstrate that EVs quickly disappear from the blood circulation and are mainly distributed to the liver after intravenous injection into mice, with a half-life of approximately 2–4 min.^163^ It is likely that surface molecules such as phospholipids and proteins are important for determining the pharmacokinetics and cellular uptake of EVs.^164^ A further question relates to the ideal route of EV administration. In this regard, a recent study demonstrated that MSC-derived EVs were effective in pigs after intramyocardial but not intracoronary delivery post reperfusion.^165^

### 5.1 Progenitor and stem cell-derived EVs

Stem cell therapy has been investigated as a potential approach to prevent cardiac damage and stimulate cardiac repair in ischaemic heart disease.^143^ Recent developments in regenerative medicine that explore the use of cell-free approaches, hence using the isolated paracrine effectors from cells, focus on EVs that are release by cultured cells.^2^^,^^81^ The use of these vesicles provide an alternative strategy for the paracrine effects of cell transplantation and activation of the repair mechanisms of the resident myocardium that captures the complexity of the signalling required to stimulate these regenerative processes.

The best studied population of cells for its paracrine effects in the heart are the mesenchymal stromal cells (MSCs). Conditioned medium of these cells reduced infarct size in both murine and porcine animal models,^166^^,^^167^ after which the exosome-containing fraction was demonstrated to be the functional fraction, which decreased oxidative stress and activated the PI3K/Akt pathway in the myocardium.^45^^,^^168^ As indicated above, several subclasses of EVs might be released by cells. A recent study showed that MSCs produce at least three subtypes of EVs, isolated based on their affinities for membrane lipid-binding ligands, and are likely to have a different biogenesis pathway and possibly different functions.^169^^,^^170^

In addition to the cardioprotective effects of EVs, pro-angiogenic effects have been observed,^170^ not only by MSCs but also by cardiac-derived progenitor cells (CPCs), which are at least partly mediated via extracellular matrix metalloproteinase inducer (EMMPRIN).^85^^,^^86^^,^^171^ In the EVs of CPCs, several miRNA clusters are enriched and important for their therapeutic effects.^82^^,^^83^ EVs derived from blood-outgrowth endothelial cells^91^^,^^172^ and especially circulating CD34^+^ stem cells^90^ were also shown to exert pro-angiogenic paracrine activity. EVs are now being reported to have anti-inflammatory effects as well, e.g. endothelial cell-derived EVs were found to suppress monocyte activation.^173^ EVs harvested from *T*_reg_ cells have been found to exert an immune-suppressive function by the suppression of monocyte activation,^174^ and EVs from MSCs can suppress T-Lymphocyte proliferation.^175^^,^^176^

Mouse embryonic stem cells secrete exosomes which can also augment neovascularization, myocyte proliferation, and survival after MI.^177^ Importantly, they were shown to augment the survival, retention, and proliferation of CPCs in ischaemic myocardium by delivery of miR-294.^177^ Exosomes have also been identified as key mediators of regeneration induced by cardiosphere-derived cells (CDCs), which they achieved partly by the transfer of miR-146a.^81^ Recently, CDC exosomes isolated by filtration and precipitation were injected into the myocardium of pigs following ischaemia and reperfusion, which reduced infarct size and improved cardiac function 4 weeks later.^165^

Recent studies described the isolation of EVs from iPSCs, with healing ability in *in vivo* model of myocardial ischaemia.^178^ Finally, plasma EVs themselves have been shown to be able to protect the myocardium from ischaemia and reperfusion injury^179^ and EVs have been shown to be necessary for endogenous cardioprotective mechanisms such as remote ischaemic conditioning.^46^

### 5.2 Hybrid nanomedicine to mimic EVs

A further approach to the use of EVs as therapeutic tools is to load them with a range of molecules or pharmacological compounds from siRNA to small molecules or proteins, serving as tool for empowering cell-derived EVs and for targeted-drug delivery.^180^ For example, liposomes with a phospholipid bilayer can carry a variety of proteins and nucleic acids as well as pharmaceutically active substances to the injured heart.^181^ Such exosome-mimetic structures can also include specific targeting molecules that enhance their targeting. Currently, liposomal transport of drug molecules are in clinical practice and under clinical trial.^182^^,^^183^ Exosome-mimetic delivery systems offer the advantage of being more controllable and scalable for clinical settings.^184^^,^^185^ Conversely, the advantage of using EVs in targeted drug delivery over the existing synthetic delivery systems (such as liposomes) includes their potentially increased circulation time, bio-distribution, decreased immunogenicity and toxicity, loading of complex cargo, cellular interactions and the different methods for therapeutic cargo loading (both post-loading and pre-loading) and administration.

### 5.3 Important limitations to overcome in the development of EV therapeutics

The use of EVs provides several advantages over the use of cells for therapeutic application. These include the absence of tumourigenicity, conservation of activity between species, lower immunogenic potential, and theoretically improved tissue-targeting potential. However, before EVs can enter the clinic as medicinal products several important steps have to be taken (*Table 3*)^43^:

**Table 3:** Current limitations for cardiac therapeutic use of EVs

(1) Unestablished regulatory aspects.
(2) Scalable production and stability of EVs.
(3) Purification problem, including potential heterogeneity of EVs and presence of co-purified molecules.
(4) Lack for standardized quality control methods for EV production.
(5) Determine which proportion of EVs mediates therapeutic effects, including unknown mode of action, including potential retention issues.
(6) Unknown pharmacokinetics of EVs as a therapeutic.
(7) Unknown safety and toxicity, including immunogenicity.
(8) Difficulty for freedom to operate due to regulatory protection issues.

1. Therapeutic differences from independent cell preparations have been observed among comparable EV fractions, which might be caused by variability between donors or *heterogeneity of EVs* harvested.^169^ Since cellular growth status varies between cell cultures, as it does for embryonic stem cells or MSCs,^186^ these differences might also be reflected in their secreted EVs.
2. Upon EV isolation and usage, contamination by *co-purified and bound molecules*might affect functional assays.^12^
3. Since isolated EVs might be heterogeneous by nature, only a *proportion* of produced EVs might be responsible for observed*therapeutic effects.*
4. The EV *pharmacokinetics* are largely unknown and need detailed follow-up in patients to understand their bio-distributions.
5. The approach of using *non-autologous EVs* in the setting of cardiovascular disease *should be taken carefully*, since EVs can potentially carry pathogen-specific antigens.
6. Currently, as it is unknown which EV fraction (content, membranes or both) is responsible for the biological activity of EVs, the *mechanism of action*should investigated.
7. Prior to entering into clinical trials, *industrial-scale, highly reproducible methods of isolating GMP-quality EVs, as well as validation procedures* are required.

## 6. Consensus statement on the steps required to take EVs forward to clinical applications as biomarkers or therapeutics

Biomarkers: As indicated in Section 4.4., the current technical limitations for EV isolation do not allow specific recommendations for the use of EVs as biomarkers, though guidelines for biomarkers in general are applicable. In the coming years, standardized pre-analytic and isolation procedures need to be further defined and simplified, including the definition of gold standards for processing, characterization, and identification of EV purity (*Table 4*). By exploring these areas, the influence of confounding factors, including co-morbidities, co-medication and whether identified EV markers are disease specific will be evaluated. The typically small yields of isolated EVs will hamper a clear full EV characterization, but technical developments that allow, for example, single cell sequencing will lower input needs for these techniques. Most important is the validation of pilot observation from small cohorts in larger dataset of patients, thereby improving the reproducibility of isolation procedures, and to provide evidence of additional value of EVs over current golden standard biomarkers. To strengthen the process of identification of EVs or EV-components as biomarkers, experiments may also be designed to identify the tissue source of the biomarker, combined with the functional validation of the identified molecule (protein and/or RNA) in the target tissues.

**Table 4:** Future perspectives: Developments required to take EVs forward to clinical applications as biomarkers or therapeutics

– Improvement of flow cytometric methods and standardization of analytical procedures. (Until this is achieved, bead-based bulk detection of EVs may provide a feasible flow cytometric detection approach irrespective of the instrument used).
– Development of novel high-resolution methodologies for EV isolation and visualizations.
– Understand mechanism of inter-cell or inter-organ communication.
– Potential source for cardiac tissue and disease specific biomarkers.
– Potential packages for cardiac specific therapies.
– Potential multi-targeting effects of EVs for the complex mechanisms of ischaemic heart disease.
– Potential advantage for EV therapeutic application over cells, including absence of tumourigenicity and cross species efficacy.

Therapy: The translation of EVs into clinical therapies will require categorization of EV-based therapeutics in compliance with existing regulatory frameworks. Since EVs will probably be considered as biological medicinal products more specifically advanced therapy medicinal products (ATMPs), new rules explicitly regulating EV-based therapies are probably not needed. However, although existing European guidance on biological active substances covers the manufacturing and clinical evaluation of novel EV-based therapeutics, special guidelines addressing EV-based therapeutics may be needed.^43^ The regulatory classification of most biological medicinal products, including ATMPs depends on a pharmaceutically active substance that does not require a molecular definition, but could mean the cells themselves in case of cellular therapeutics.^187^ Here, one needs to identify, quantify, and characterize the main effector that is causing the biologic effect and define the mode or mechanism of action.

Similar to human cell-based products,^188^ preclinical safety testing might be needed as well as a risk-analysis approach for the transition from preclinical to clinical development, thereby taking into account the heterogeneity among independent cell donors and different preparations, as well as the EV heterogeneity in obtained donor cell samples. A test for biological activity should be included unless otherwise justified. However, since many cell products are composed of a heterogeneous mixture of cells, identification of the cellular component(s) responsible for a proposed biological activity will be a major challenge. General clinical requirements and developments have been recently reviewed for cardiac cellular therapeutics.^143^

Based on the legislation for ATMPs including tissues, cells, a set of minimal criteria for the characterization of such products needs to be considered before use in clinical trials. One needs to define whether a product is (i) of autologous, allogeneic or xenogeneic origin; (ii) extensively or minimally manipulated *in vitro*; and (iii) immunologically active or neutral, (iv) is the proliferative capacity of cells and tissue-like organization, as well as defining (v) the dynamic interaction between these elements, (vi) the intended use.^43^^,^^189^

In summary, to advance towards first-in-man-clinical trials approval of the technical requirements and quality risk management is needed, donor safety, recipient safety, and release criteria for EV-based therapeutics should be defined and quality control should be in place, including molecular and physical EV characterization, and *in vitro* potency assays. Moreover, the identified recommendation criteria for the characterization of isolated EV are critical for both their therapeutic use and for biomarker discovery.

### Funding

JS is supported by Horizon2020 ERC-2016-COG EVICARE (725229), the Project SMARTCARE-II of the BioMedicalMaterials Institute, co-funded by the ZonMw‐TAS program (#116002016), the Dutch Ministry of Economic Affairs, Agriculture and Innovation and the Netherlands CardioVascular Research Initiative (CVON): the Dutch Heart Foundation, Dutch Federations of University Medical Centers, the Netherlands Organization for Health Research and Development, and the Royal Netherlands Academy of Sciences. SD is supported by Project grant PG/16/85/32471 from the British Heart Foundation. PF and RS were supported by the European Cooperation in Science and Technology (COST EU-ROS BM1203). DH is the chair, PF is the vice chair, and SD and RS are working group leaders of COST Action EU-Cardioprotection (CA16225). PF holds grants from the Hungarian National Research, Development, and Innovation Office (OTKA K 109737, OTKA ANN 107803, NVKP 16-1-2016-0017, and VEKOP-2.3.2-16-2016-00002). EIB is supported by NVKP 16-1-2016-0017, OTKA 20237, OTKA 111958, and VEKOP-2.3.3-15-2017-00016. RK is supported by National Institutes of Health Grants HL091983, HL126186 and HL134608. CP is supported by the Italian Ministry of University, Research and Technology (RBFR124FEN and 2015583WMV grants) and by Programme STAR, financially supported by Compagnia di San Paolo and Federico II University, Naples (Italy). SL holds grant from the South African National Research Foundation. FBE is supported by the German Research Foundation (DFG Research Unit FOR2149). DdK is supported by Queen of Hearts Program Dutch Heart Foundation 2013T084; BMRC CS-IRG, ATTRaCT SPF grant. CMB is supported by Insitut National de la Santé et de la Recherche Medicale, Université Paris Descartes and ANR-16-CE92-0032-02. DH was supported by the British Heart Foundation (FS/10/039/28270), Duke-National University Singapore Medical School, the National Institute for Health Research University College London Hospitals Biomedical Research Centre, Singapore Ministry of Health's National Medical Research Council under its Clinician Scientist-Senior Investigator scheme (NMRC/CSA-SI/0011/2017) and Collaborative Centre Grant scheme (NMRC/CGAug16C006), the Singapore Ministry of Education Academic Research Fund Tier 2 (MOE2016-T2-2-021), and the National Research Foundation (NRF) Singapore (NRF-CRP13-2014-05).

**Conflict of interest**: PF is the founder and CEO of Pharmahungary Group, a group of R&D companies.
